# Identifying Existing Guidelines, Frameworks, Checklists, and Recommendations for Implementing Patient-Reported Outcome Measures: Protocol for a Scoping Review

**DOI:** 10.2196/52572

**Published:** 2024-05-21

**Authors:** Randi Thisakya Jayasinghe, Susannah Ahern, Ashika D Maharaj, Lorena Romero, Rasa Ruseckaite

**Affiliations:** 1 School of Public Health and Preventive Medicine Monash University Melbourne Australia; 2 The Ian Potter Library Alfred Hospital Melbourne Australia

**Keywords:** patient-reported outcome measures, patient-reported outcomes, quality of life, clinical quality registry, guidelines, frameworks, recommendations, scoping review, patient perspectives, patient perspective, patient-reported outcome, patient-reported, clinical setting, clinical registry, registry, systematic review

## Abstract

**Background:**

Implementing patient-reported outcome measures (PROMs) to measure and evaluate health outcomes is increasing worldwide. Along with this emerging trend, it is important to identify which guidelines, frameworks, checklists, and recommendations exist, and if and how they have been used in implementing PROMs, especially in clinical quality registries (CQRs).

**Objective:**

This review aims to identify existing publications, as well as publications that discuss the application of actual guidelines, frameworks, checklists, and recommendations on PROMs’ implementation for various purposes such as clinical trials, clinical practice, and CQRs. In addition, the identified publications will be used to guide the development of a new guideline for PROMs’ implementation in CQRs, which is the aim of the broader project.

**Methods:**

A literature search of the databases MEDLINE, Embase, CINAHL, PsycINFO, and Cochrane Central Register of Controlled Trials will be conducted since the inception of the databases, in addition to using Google Scholar and gray literature to identify literature for the scoping review. Predefined inclusion and exclusion criteria will be used for all phases of screening. Existing publications of guidelines, frameworks, checklists, recommendations, and publications discussing the application of those methodologies for implementing PROMs in clinical trials, clinical practice, and CQRs will be included in the final review. Data relating to bibliographic information, aim, the purpose of PROMs use (clinical trial, practice, or registries), name of guideline, framework, checklist and recommendations, the rationale for development, and their purpose and implications will be extracted. Additionally, for publications of actual methodologies, aspects or domains of PROMs’ implementation will be extracted. A narrative synthesis of included publications will be conducted.

**Results:**

The electronic database searches were completed in March 2024. Title and abstract screening, full-text screening, and data extraction will be completed in May 2024. The review is expected to be completed by the end of August 2024.

**Conclusions:**

The findings of this scoping review will provide evidence on any existing methodologies and tools for PROMs’ implementation in clinical trials, clinical practice, and CQRs. It is anticipated that the publications will help us guide the development of a new guideline for PROMs’ implementation in CQRs.

**Trial Registration:**

PROSPERO CRD42022366085; https://tinyurl.com/bdesk98x

**International Registered Report Identifier (IRRID):**

DERR1-10.2196/52572

## Introduction

Patient-reported outcome measures (PROMs) are standardized, validated questionnaires that seek to ascertain patients’ views on their physical and mental well-being [1]. Traditionally, PROMs were developed for use in research, to assess treatment effectiveness in clinical trials, and were later adopted by clinicians to enhance their clinical management of patients [2,3]. PROMs are increasingly accepted to measure outcomes of an intervention from the patient’s point of view in various health care and research settings including clinical practice, clinical trials, and clinical quality registries (CQRs) [1,4,5].

PROMs are routinely used in clinical trials to assess the impact of treatment from the patient’s perspective, especially in disease-specific areas such as oncology and cardiovascular disease [6,7]. For instance, the Australian government supports a Quality of Life Office that collaborates with 13 National Cancer Clinical Trial Groups to include PROs in investigator-initiated oncology trials such as breast cancer trials [6,8]. A review of the Australian New Zealand Clinical Trials Registry indicated that of 13,666 trials registered from 2005 to 2017 in the registry, 6168 (45%) included a PROM [9].

PROMs in clinical trials are important for decision-making among the stakeholders involved, especially in medical device-related trials to measure adverse events and other negative outcomes to assess the safety of a medical device [10]. Evidence indicates that in Western European nations such as the United Kingdom and Germany, the use of PROMs in medical device studies is increasing [11]. The European Medicines Agency also has a guideline on the use of patient-reported outcomes (PROs) in evaluating anticancer medical products [12].

In countries such as Australia, there are national-level initiatives, such as the National Safety and Quality Health Service standards, that encourage the involvement of patients in planning, delivering, monitoring, and evaluating health care [13]. The use of PROMs in clinical practice has increased over the years as a collection of PROs “ensures the patient’s values and expectations are present in all aspects of care ensuring management remains patient-centred” [14,15]. For instance, organizations such as the International Consortium for Health Outcomes Measurements drive the use of PROMs in clinical practice [16]. At the patient level, PROMs may be used for screening, monitoring, promoting patient-centered care, and facilitating better communication between the patient and the provider. At a higher level, PROMs may be used to facilitate system health care planning and assess the quality of health care delivery over time [17,18].

CQRs systematically monitor the quality of health care within specific clinical domains by routinely collecting, analyzing, and reporting health-related information to their numerous stakeholders [13]. CQRs have expanded in the last decade to monitor clinical outcomes and facilitate evidence-based clinical practice in most developed countries [5,19,20]. Many registries also capture PROs on the expectations and impact of treatment from patients to provide benchmark reports for quality improvement within clinical settings [21].

PROMs captured in clinical practice, clinical trials, and CQRs can contribute to understanding variation in health care, efficacy, and safety of interventions and potentially improve health care provision. Implementation of these measures, however, is complex, and requires a thorough consideration of various aspects such as selecting the appropriate PROM, determining the mode and method of administration, ensuring good quality data are captured and high response rates are achieved, and analysis and reporting of the data [22]. Along with the increase in the use of PROMs, it is important to ensure that guidelines, frameworks, checklists, and recommendations for implementing PROMs, addressing various aspects of PROMs’ implementation, exist to aid successful PROs collection.

The findings of this scoping review will help us identify which guidelines, frameworks, checklists, and recommendations exist and if and how they have been applied in PROMs’ implementation in clinical trials, clinical practice, and CQRs. In addition, the publications identified will help us create a preliminary set of recommendations that can be used when developing a new guideline for PROMs’ implementation in CQRs.

## Methods

### Information Sources and Search Strategy

A comprehensive literature search will be performed using a search strategy developed by a medical librarian (LR). The search will be performed using both MeSH (Medical Subject Headings) terms and free text including PROMs AND (“clinical trials” OR “clinical practice” OR “clinical quality registry”). The detailed search is provided in Multimedia Appendix 1.

The review will include MEDLINE, Embase, CINAHL, PsycINFO, and Cochrane Central Register of Controlled Trials databases. The publications will be limited to those in the English language. All publications from database searches will be exported to Covidence (Veritas Health Innovation) [23], a software that assists the paper screening, database extraction, and cooperation among multiple assessors, and duplicates will be removed for screening.

The protocol for this scoping review has been developed according to the PRISMA-P (Preferred Reporting Items for Systematic Review and Meta-Analysis Protocols) guideline or checklist [24].

### Selection and Screening Process

Two independent researchers (RTJ and RR) will conduct title and abstract screening of all records including publications of guidelines, frameworks, checklists, recommendations, conference abstracts, opinion pieces, websites, media articles, and presentations identified in the initial search to determine eligibility. The eligibility will be determined according to the inclusion and exclusion criteria described below. The last phase will include 3 researchers (RTJ, RR, and ADM) independently reviewing full-text papers to determine eligibility for the review. Any conflicts will be resolved through discussion and consensus.

### Inclusion Criteria

Publications describing the implementation of PROMs in any clinical setting for various purposes including clinical trials, clinical practice, and CQRs will be included in the final review. The included publications must describe the conception, development, or application of the guidelines, frameworks, checklists, and recommendations for implementing PROMs. PROMs’ implementation will include all aspects from selecting the appropriate PROM, integrating it into practice, collection of the data, analysis, and reporting. Qualitative, quantitative, and mixed methods studies will be included. Government and clinical registry reports and gray literature including guidelines, frameworks, checklists, and recommendations documents and websites will also be included.

### Exclusion Criteria

Non-English publications and any unpublished papers, dissertations, conference proceedings, and meeting abstracts will be excluded.

### Data Extraction

Data extraction will be independently performed by the 3 reviewers (RTJ, RR, and ADM), and any discrepancies in the data will be resolved through discussion and consensus with the senior author. Evidence will be synthesized based on the information presented in Textbox 1.

**Data extraction items of included publications.**

**Bibliographic information**

- Author, year, and country of publication
- Title

**Publications of guidelines, framework, checklists, and recommendations**

- Name of the guideline, framework, checklist, and recommendations
- Purpose of patient-reported outcome measures (PROMs) use (ie, clinical trial, practice, or registry)
- Aspects or domains of PROMs’ implementation addressed by the guideline, framework, checklist, and recommendations (ie, selection of the tool, administration, data collection, analysis, and reporting)

**Publications discussing the use and application of guidelines, framework, checklists, and recommendations**

- Brief aims of the study
- Purpose of PROMs use (ie, clinical trial, practice, or registry)
- Condition (disease)
- Name of the guideline, framework, checklist, and recommendations
- Rationale for development and purpose of the guideline, framework, checklist, or recommendations (ie, development and implementation of PROMs including selection of the tool, administration, data collection, analysis, and reporting)
- Implications of applying the guideline, framework, checklist, and recommendations for PROMs implementation (ie, changes in clinical practice, improving outcomes, and response rates)

### Data Management

The search will be carried out in the databases mentioned in the Methods section under “Information Sources and Search Strategy” and then loaded into EndNote X8 software (Clarivate) [25], a management software for references that allows the identified references to be organized into different electronic databases. All results will be inserted in a single EndNote folder and duplicated publications will be identified and removed using EndNote X8 [25]. After duplicate removal of publications, the research results will be loaded onto Covidence [23].

### Data Analysis

A narrative synthesis of the data will be conducted using tables and texts in this scoping review. A table with a summary of study characteristics from the data extracted will be presented. This will indicate existing guidelines, frameworks, checklists, and recommendations for implementing PROMs in any clinical setting, their purpose, and the implications. For the analysis, data will be grouped according to the purpose of PROMs use (ie, clinical trials, clinical practice, or CQRs). The narrative synthesis will be conducted by summarizing and explaining the extracted data from Textbox 1. This will include discussing the rationale for the development and implications of existing guidelines, frameworks, checklists, and recommendations for different purposes. The need for an appropriate guideline in CQRs will be discussed as well.

### Ethical Considerations

Ethical approval is not required for this scoping review because all the data included in the review has been published and is publicly available. This review did not include the collection of data from human participants.

## Results

The review was registered in PROSPERO on October 21, 2022 (CRD42022366085). Electronic database searches have been completed in March 2024, title and abstract screening, full-text screening, as well as data extraction will be completed in May 2024. As of May 2024, a total of 4905 records have been identified, 177 articles have been assessed for eligibility and 56 studies have been selected for data extraction. The study is expected to be completed by the end of August 2024.

## Discussion

### Principal Findings

The proposed scoping review aims to identify any existing guidelines, frameworks, checklists, and recommendations in clinical trials, practice, and registries. We anticipate that the findings will also guide the development of a new guideline for PROMs’ implementation in CQRs.

This review will be the first part of a broader project to develop a guideline for PROMs’ implementation in CQRs. The findings will help us identify existing guidelines, frameworks, checklists, and recommendations and various aspects of the PROMs’ implementation they address. These may include PROMs’ selection, administration, collection, analysis, and reporting. Furthermore, the review will provide evidence of how the guidelines, frameworks, checklists, and recommendations have been developed and their implications (including barriers and enablers) when used in PROMs’ implementation in clinical trials, clinical practice, and CQRs. From these findings, we will be able to create a set of preliminary recommendations that can be used as the foundation to guide the development of a new guideline for PROMs’ implementation in CQRs.

In addition to clinical trials and practices, clinical registries also use PROMs, especially for reporting and benchmarking purposes [1]. The significance of PROMs’ data from CQRs is that they can be collected from a large population and outcomes can be monitored over a long time. Additionally, the collection of PROMs in CQRs has many implications. These include improving patient outcomes, improving quality of care, improving response rates, minimizing the cost and resource burden, and providing benchmarks for participating health services. Despite these benefits, no standard guideline, framework, checklist, and recommendations exist for the implementation of PROMs in CQRs.

Having a standard guideline, framework, checklist, and recommendations to collect patients’ perspectives appropriately will be useful for CQRs in many different ways. For instance, long-term monitoring of a patient’s condition through PROMs helps clinicians understand the status of a patient’s health during a disease or treatment [22]. This can facilitate patient-clinician communication and support making informed decisions regarding treatment options based on the patient’s opinion. However, these opinions will only be useful if they are collected, analyzed, and reported in a useful manner. Hence, a standard guideline outlining the steps to implement PROMs will ensure that patient perspectives are captured successfully.

Thus, by using the findings from this review as the foundation, followed by a consensus study, validation, and testing studies, our ultimate aim is to develop a guideline for PROMs’ implementation in CQRs worldwide.

### Limitations

There is potential for heterogeneity in the papers included in this review. Also, the reproducibility of the study might be limited.

### Conclusions

Implementation of PROMs is complex and requires a thorough consideration of various aspects such as selecting the most appropriate PROM, determining mode and method of administration, ensuring good quality data are captured and high response rates are achieved, and analysis and reporting of the data. This review findings will help identify existing guidelines, frameworks, checklists, and recommendations in clinical trials, clinical practice, and CQRs. The findings will also guide the development of a new guideline for PROMs’ implementation in CQRs.

## Appendix

Multimedia Appendix 1 Search strategy of the Ovid MEDLINE database.

## Abbreviations

CQR: clinical quality registry
MeSH: Medical Subject Headings
PRISMA-P: Preferred Reporting Items for Systematic Review and Meta-Analysis Protocols
PRO: patient reported outcome
PROM: patient-reported outcome measure
