# Supplementary material for: Identifying Existing Guidelines, Frameworks, Checklists, and Recommendations for Implementing Patient-Reported Outcome Measures: Protocol for a Scoping Review
Source: JMIR Res Protoc. 2024 May 21;13:e52572. doi: 10.2196/52572 (PMC11150888; doi:10.2196/52572)
Supplement: Multimedia Appendix 1 [file resprot_v13i1e52572_app1.docx]

| **#** | **Query** |
| --- | --- |
| 1 | patient reported outcome measures/ |
| 2 | ((patient or self) adj (reported outcome* or reported treatment*)).mp. |
| 3 | ((patientreported or selfreported) adj (outcome* or treatment*)).mp. |
| 4 | self reported treatment outcome*.mp. |
| 5 | 1 or 2 or 3 or 4 |
| 6 | Registries/ |
| 7 | clinical quality regist*.mp. |
| 8 | (registr* or register*).mp. |
| 9 | clinical trial*.mp. |
| 10 | clinical practice*.mp. |
| 11 | clinical regist*.mp. |
| 12 | clinical database*.mp. |
| 13 | 6 or 7 or 8 or 9 or 10 or 11 or 12 |
| 14 | framework*.mp. |
| 15 | recommendation*.mp. |
| 16 | guideline*.mp. |
| 17 | (checklist* or check list*).mp. |
| 18 | 14 or 15 or 16 or 17 |
| 19 | 5 and 13 and 18 |
